# Prevalence of visual impairment and outcomes of cataract surgery in Chaonan, South China

**DOI:** 10.1371/journal.pone.0180769

**Published:** 2017-08-10

**Authors:** Xiujuan Zhang, Emmy Y. Li, Christopher Kai-Shun Leung, David C. Musch, Xin Tang, Chongren Zheng, Mingguang He, David F. Chang, Dennis Shun-Chiu Lam

**Affiliations:** 1 Department of Ophthalmology and Visual Sciences, the Chinese University of Hong Kong, Hong Kong, China; 2 Tianjin Eye Hospital, Tianjin, China; 3 Project Vision Charitable Foundation, Hong Kong, China; 4 Hong Kong Eye Hospital, Kowloon, Hong Kong, China; 5 Department of Ophthalmology and Visual Sciences, University of Michigan Medical School, Ann Arbor, Michigan, United States of America; 6 State Key Laboratory of Ophthalmology, Sun Yat-Sen University, Guangzhou, China; 7 The University of California, San Francisco, California, United States of America; National Eye Institute, UNITED STATES

## Abstract

**Purpose:**

To estimate the prevalence and causes of blindness and visual impairment (VI), and report the outcomes of cataract surgery in Chaonan Region, Guangdong Province, southern China

**Design:**

Cross-sectional population-based survey

**Participants:**

A total of 3484 participants including 1397 men (40.1%) and 2087 women (59.9%) aged ≥50 years were examined (94.2% response rate).

**Method:**

A two-stage cluster sampling procedure was used to select 3700 participants aged ≥50 years from 74 clusters of Chaonan Region. Participants were examined according to the Rapid Assessment of Avoidable Blindness (RAAB) method. Blindness and visual impairment (VI) were defined by the World Health Organization criteria. Participants with visual acuity (VA) < 6/18 in either eye were examined by ophthalmologists. The primary causes of blindness and VI were reported with reference to the participant’s better eye.

**Main outcome measures:**

Prevalence and main causes of blindness, severe visual impairment (SVI), VI and the outcomes of cataract surgery

**Results:**

The standardized prevalence rates of blindness, SVI, and VI were 2.4% (95% confidence interval [CI], 1.9–2.9%), 1.0% (95% CI, 0.7–1.4%), and 6.4% (95% CI, 5.6%– 7.1%), respectively. The principal cause of blindness and SVI was cataract, accounting for 67.1% and 67.6% respectively, and the principal cause of VI was refractive error (46.9%). One hundred and fifty five out of 3484 (4.4%) people (211 eyes) had cataract surgery. Of the 211 eyes that had cataract surgery, 96.7% were pseudophakic. 67.2% of the 211 operated eyes had a presenting visual acuity (PVA) of 6/18 or better.

**Conclusions:**

The prevalence of blindness, SVI, and VI was high among rural residents in Chaonan. Cataract remained the leading cause of avoidable blindness. Outcomes of cataract surgery performed in rural private clinics were suboptimal. Quality-control initiatives such as hands-on training program should be introduced to improve cataract surgery outcomes.

## Introduction

In 2010, the World Health Organization (WHO) estimated that there were 39 million people in the world with blindness and 285 million with visual impairment. China had the largest number with 8 million blind and 75 million visually impaired individuals[1]. Population based surveys in China have identified cataract as the leading cause of blindness and visual impairment[2–7]. Cataract surgery is an effective means to reverse cataract blindness. However, the cataract surgical rate (CSR) in China remains relatively low at about 772 cases per one million per year,[8] which is much lower than its neighbor India[9]. A major cause of the low CSR is the lack of experienced surgeons in rural areas. The Chinese Ministry of Health has reported that 45% of China’s 2400 county hospitals do not offer cataract surgery services[10] and most rural residents are unable to afford surgery in urban centers. Thus, a large number of patients with severe cataract have little or no access to affordable surgical services. On this account, Project Vision, a non-governmental organization, was established to build a sustainable model to reduce cataract blindness in rural China. Project Vision’s first priority is to develop rural charity eye centers wherein local doctors are trained to provide high quality and low cost cataract surgery[11].

Liangying Hospital, located in the Chaonan Region, was the first county hospital designated to be a local charity eye center in 2004. After 6 months of hands-on training, the local surgeons were approved to perform cataract surgery independently by at least two experienced senior ophthalmologists from Project Vision[12, 13]. The number of cataract operations increased annually in the past seven years. The two surgeons have performed approximately 11,000 cataract operations since the establishment of the Charity eye center in 2004.

Rapid Assessment of Avoidable Blindness (RAAB) aims to provide information on the prevalence of visual impairment due to avoidable causes of vision loss[14]. RAAB also collects data on cataract surgical coverage, major barriers to the uptake of cataract surgery, and visual outcome after surgery for individuals aged ≥50 years. The aim of this study was to define the prevalence of blindness and visual impairment among people aged ≥50 years in Chaonan and to evaluate the outcomes of cataract surgery performed in the region.

## Methods

This study was approved by the Ethics Committee of the Chinese University of Hong Kong and the Disabled Federation of Chaonan Region, and conformed to the tenets of the Declaration of Helsinki. Subjects aged ≥50 years, residing in 11 towns and 241 villages in the Chaonan region were enrolled from April to July in 2012. Persons were considered ineligible if they had moved out of the village, had not lived there in the past 6 months since we selected the sample in April 2012. All the study procedures were explained in detail to each subject and the family by the study investigators with local dialect, in the presence of two to three community heads of the village. Written informed consent with signature or inked fingerprint was obtained from all participants before examination.

### Cataract surgical facilities in research setting

The study site, Chaonan Region, is a rural plane, situated in eastern Guangdong, approximately 150 km from the provincial capital of Guangzhou City and 36 km from Shantou City. The Zhongshan Ophthalmic Center (ZOC) and the Joint Shantou International Eye Center (JSIEC), located in Guangzhou and Shantou, respectively, are two major public hospitals in China. There were eighteen experienced surgeons providing cataract surgery in ZOC, and six surgeons in JSIEC. Besides the two public hospitals, there are other facilities that can provide cataract surgeries in the local area.

1. Charity eye centers: There were three surgeons performing cataract surgeries in two charity eye centers of this region. As these local surgeons became recognized by residents in the area, the number of patients receiving cataract surgery in charity centers steadily increased.
2. Private eye clinics: There were three private eye clinics with four surgeons providing cataract surgery services in the region.

### Sample size calculation

According to the 2010 Chaonan Region Census, the population size of Chaonan Region was 1,288,165, and the target population aged ≥50 years was 214,425[15]. According to the report from the China Nine-province Survey in 2008, the expected prevalence of blindness in adults aged 50 or above was estimated to be 2.68% in Guangdong Province[16]. With a confidence interval of 95%, a precision of 25%, and a design effect set at 1.5, the minimum sample size was 3,295. Assuming a 90% response rate; the sample size was estimated to be 3,662. We therefore selected 74 clusters, each with 50 participants.

### Selecting the sample

A two stage sample selection was adopted[14, 17]. A cluster random sampling with probability proportional to population size, as proposed by Wu et al[5], was performed to select 74 clusters. In brief, names of the enumeration area and the corresponding cumulative population were listed to create a sampling frame. The sampling interval was calculated as the total population divided by the number of clusters required (i.e., 74). The starting point was first determined by multiplying a computer-generated random number between 0 and 1 with the sampling interval. The starting point was then tracked in the cumulative column and the corresponding enumeration area was selected as the first cluster. The second cluster was identified by adding the sampling interval to the starting point number and the same procedures were repeated until 74 enumeration areas were selected. An enumeration area is either a large village with a population of more than 1000 or a suburb residence at administrative level composed by a number of small natural villages, each with around 150–200 residents, defined by geographical boundaries. The natural villages were considered as segments in the enumeration area and numbered. For each selected enumeration area, all the segment numbers were written on small pieces of paper and one was randomly picked.

In each segment, the random walk method was used to enroll the first 50 people aged 50 years and above. A random direction was chosen prior to the survey. After starting from a randomly selected household, the next nearest household in the direction chosen was visited until 50 subjects aged 50 or above were identified[4, 14]. Those available and willing were examined on-site. If there were households who were known to be residents aged 50 or above, but unavailable at the time of the first visit, the survey team would revisit on another day to examine them. When all the households in a selected segment were visited and there were less than 50 eligible subjects, the next nearest segment in the enumeration area were visited to complete the cluster.

### Training

A 7-day workshop was held under the supervision of two expert ophthalmologists who had conducted RAAB surveys prior to commencement of this survey. Two research teams including ophthalmologists, optometrists, computer operators and local research assistants conducted the surveys. Training and inter-observer variation test were performed 3 weeks before the commencement of the survey. Each team examined the same group of subjects during a pre-study pilot according to the protocol described in the RAAB manual. These subjects were examined by 3 ophthalmologists and the examination findings were compared to the most experienced examiner in the team, Dr ZXJ, to calculate the inter-observer agreement. The assessment included examination of visual acuity, lens status, and tests to determine the main cause of visual loss. Kappa for agreement between teams on examination findings was calculated during the pre-study pilot, and a kappa of at least 0.80 was required before the survey commenced. During the first 2 weeks, the two research teams worked together to examine 50 people in the same cluster under supervision to minimize discrepancy between different examiners. After that, a field supervisor (ZXJ) accompanied the teams at least once a week during the study to check the reliability and validity of data collection.

### Definitions

According to the World Health Organization (WHO) definition, blindness was defined as visual acuity (VA) of <3/60, severe VI (SVI) was defined as VA ≥3/60 and <6/60 and visual impairment (VI) was defined as VA≥6/60 and <6/18 in the better eye with available spectacle correction. Refractive error, a cause of visual impairment, was defined as VA<6/18 that improved to ≥6/18 with a pinhole. Cataract was defined as visible opacity in the pupil impairing vision with partial or complete obscuration of red reflex on distant direct ophthalmoscopy. Operated eyes with cataract surgery were defined as all pseudophakic/aphakic eyes due to cataract. Cataract surgical coverage (CSC) percentage for persons was calculated as: Number of persons operated in either eye × 100/Number of persons operated + Number of persons bilaterally visually impaired by cataract (Pinhole VA<3/60, <6/60 or <6/18). The CSC percentage for eyes was calculated as follows: Number of operated eyes × 100 /Number of operated eyes + Number of operable cataract eyes (Pinhole VA<3/60, <6/60 or <6/18)[18]. The causes of blindness and visual impairment were classified into 4 groups: curable (cataract, refractive error, uncorrected aphakia); preventable (surgical complications, trachoma, phthisis, other corneal scars, onchocerciasis); potentially preventable (diabetic retinopathy, glaucoma); and other causes (age-related macular degeneration (AMD), posterior segment lesions, central nervous system defects). The first 2 categories (preventable and curable) were designated as “avoidable causes”[19].

### Ophthalmic examination

Visual acuity was measured by a research assistant at the household, using a tumbling “E” letter with optotype size 6/18 on one side and 6/60 on the other side at 6 and 3 m. All measurements were taken in full daylight with available spectacle correction. If the VA with available correction was <6/18 in either eye, then pinhole vision would be measured following the same procedure. For assessment of lens status, distant direct ophthalmoscope was used in a shaded or semi-dark environment without pupillary dilatation. Relative afferent pupillary defect (RAPD) was checked with a swinging flashlight test and the fundus was examined using a direct ophthalmoscope by the ophthalmologist in all participants with VA<6/18 in either eye[5]. The pupils were dilated with a short-acting 1% tropicamide eye drops to confirm the cause of visual impairment when the pinhole VA was <6/18. The principal cause of blindness or VI was recorded by the ophthalmologists. When there were co-existing primary disorders in the same or different eyes, mark as the principal disorder that which is most readily curable. The following is a recommended ranking of the disorders with respect to these criteria by RAAB protocol: refractive error, cataract, uncorrected aphakia, surgery related complications, preventable corneal opacities and phthisis, glaucoma, and other posterior segment disorders.

A mobile clinic equipped with simple instruments including slit lamp and indirect ophthalmoscope was set up in the same village where the research team conducted the RAAB survey. Subjects were advised to attend the clinic for further examination if a definitive diagnosis could not be made. Tropicamide 1% eye drops were instilled to check for the posterior segment pathology and to define the cause of visual impairment.

### Statistical analysis

A data entry package, EpiData Entry 3.1(freeware, http://www.epidata.dk), was used for this study, which incorporated range and consistency checks. Data were entered independently by two operators in two separate computers. Consistency checks were performed each afternoon and inconsistencies were adjudicated on the same day[20].

Prevalence of blindness, SVI and VI were estimated, and the effect of cluster sampling was taken into account by using the “proc survey” commands in SAS statistics software version 9.3 (SAS Institute Inc, Cary, NC) to allow for the cluster sampling approach for calculation of prevalence, odds ratio (OR) and 95% confidence intervals (CIs)[20, 21]. Age and gender adjusted prevalence were calculated with reference to the National Population census 2010 data in Chaonan Region. Visual outcome of cataract surgery was reported among all the pseudophakic or aphakic eyes. Cataract surgery coverage was calculated by eye and person.

## Results

### Study participation

Among the 3700 people selected from 74 clusters, 169 (4.6%) could not be reached, 20 (0.5%) were physically or mentally unable to communicate, and 27 (0.7%) refused to be examined. Consequently, 3484 persons (response rate 94.2%) with a mean (±standard deviation) age of 64.4±9.8 years were examined. These included 1397 men (40.1%) with an average age of 65.3±9.6 years and 2087 women (59.9%) with an average age of 63.8±9.8 years. A comparison of the age and gender distributions between the sampled and total populations is shown in Table 1.

**Table 1:** Age and gender distribution of the sample compared to the total population in the survey area.

Age group (yrs)	Male						Female						Total					
	Census		Survey		Sample		Census		Survey		Sample		Census		Survey		Sample	
	n	(%)	n	(%)	n	(%)	n	(%)	n	(%)	n	(%)	n	(%)	n	(%)	n	(%)
**50–59**	53892	(52.0)	583	(37.7)	502	(36.0)	52140	(46.3)	975	(45.3)	948	(45.4)	106032	(49.4)	1558	(42.1)	1450	(41.6)
**60–69**	26073	(25.2)	505	(32.6)	461	(33.0)	27583	(24.5)	616	(28.6)	602	(28.8)	53656	(25.0)	1121	(30.2)	1063	(30.5)
**70–79**	17271	(16.7)	337	(21.8)	316	(22.6)	18831	(16.7)	365	(17.0)	361	(17.3)	36102	(16.8)	702	(19.0)	677	(19.4)
**>80**	6424	(6.2)	123	(7.9)	118	(8.4)	12211	(10.9)	196	(9.1)	176	(8.4)	18635	(8.7)	319	(8.6)	294	(8.4)
**Total**	103660	(100.0)	1548	(100.0)	1397	(100.0)	110765	(100.0)	2152	(100.0)	2087	(100.0)	214425	(100.0)	3700	(100.0)	3484	(100.0)

### Prevalence of blindness and visual impairment

As shown in Table 2, the unadjusted prevalence in the better seeing eye of blindness, SVI and VI in the examined population aged ≥ 50 year were 2.4% (95% CI, 1.9%–2.9%), 1.1% (95% CI, 0.7–1.4%), and 7.0% (95% CI, 6.2%–7.8%), respectively. Rates for males were lower than for females for all three categories of impairment. Age and gender adjusted prevalence was 2.4% (95% CI, 1.9%– 2.9%), 1.0% (95% CI, 0.7–1.4%), and 6.4% (95% CI, 5.6%– 7.1%), respectively (Table 2). For persons with PVA (presenting visual acuity)<6/18 in the better eye, the unadjusted prevalence was 10.5% (95% CI, 9.5–11.5%) and the age and gender adjusted prevalence was 9.8% (95% CI, 8.8–10.8%), respectively. The prevalence of blindness based on pin-hole VA decreased to 2.2% (77 people, 95%CI, 1.7–2.7).

**Table 2:** Prevalence of blindness, severe visual impairment, and visual impairment in the better seeing eye.

	Male					Female					Total				
	*No*.	Crude prevalence		Standardized prevalence		*No*.	Crude prevalence		Standardized prevalence		*No*.	Crude prevalence		Standardized Prevalence	
		%	(95% CI)	%	(95% CI*)		%	95% CI	%	(95% CI*)		%	95% CI	%	(95% CI^+^)
**Blindness**	24	1.7	(1.3–2.1)	1.5	(0.9–2.1)	61	2.9	(2.3–3.5)	3.3	(2.6–4.1)	85	2.4	(1.9–2.9)	2.4	(1.9–2.9)
**SVI**	13	0.9	(0.6–1.2)	0.8	(0.3–1.2)	24	1.2	(0.8–1.5)	1.3	(0.8–1.8)	37	1.1	(0.7–1.4)	1.0	(0.7–1.4)
**VI**	79	5.7	(4.9–6.4)	4.5	(3.5–5.4)	164	7.9	(7.0–8.8)	8.2	(7.0–9.4)	243	7.0	(6.2–7.8)	6.4	(5.6–7.1)
**Any visual impairment**	116	8.3	(7.4–9.2)	6.7	(5.5–7.9)	249	11.9	(10.8–13.0)	12.8	(11.4–14.1)	365	10.5	(9.5–11.5)	9.8	(8.8–10.8)

VI: visual impairment, was defined as presenting visual acuity <6/18 and > = 6/60. SVI: severe visual impairment, defined as presenting visual acuity <6/60 and > = 3/60. CI: confidence interval

*: Age standardized to the population of Chaonan Region in 2010 National Population Census.

^+^: Age and gender standardized to the population of Chaonan Region in 2010 National Population Census.

### Risk factors for blindness and visual impairment

Of 3484 subjects, 134 subjects refused to give us the information about their literacy and marital status (S1 Table). Consequently, 3350 persons were interviewed for risk factors of blindness and visual impairment. Gender, literacy and marital status-adjusted logistic regression analyses (Table 3) revealed that the prevalence of blindness increased significantly, with subjects in the 80+ year age group carrying 15.6-fold (95% CI, 6.7–36.2) higher odds of blindness than subjects aged 50 to 59 years. The odds of blindness was lower among those who were literate (OR, 0.6; 95% CI, 0.4–1.1) with adjusted by other factors. Participants who were widowed or unmarried had more than twice the odds of blindness compared with the married (OR, 2.4; 95% CI, 1.4–4.0). Similar patterns were observed for SVI, and VI (Table 3).

**Table 3:** Blindness, severe visual impairment, and visual impairment by age, gender, education and marriage status.

	Blindness				SVI				VI				Any visual impairment			
	*No*.	(%)	Adjusted OR (95% CI)		*No*.	(%)	Adjusted OR (95% CI)		*No*.	(%)	Adjusted OR (95% CI)		*No*.	(%)	Adjusted OR (95% CI)	
**Age**+ **(yrs)**																
50–59	9	(0.6)	1		2	(0.1)	1		35	(2.5)	1		46	(3.3)	1	
60–69	9	(0.9)	1.2	(0.5–3.1)	5	(0.5)	4.0	(0.8–21.1)	58	(5.7)	2.5	(1.6–3.8)	72	(7.7)	2.3	(1.6–3.4)
70–79	25	(3.8)	4.7	(2.1–10.6)	11	(1.7)	11.4	(2.4–54.4)	81	(12.3)	6.0	(3.9–9.3)	117	(17.8)	6.4	(4.4–9.3)
>80	33	(12.2)	15.6	(6.7–36.2)	14	(5.2)	27.3	(5.5–135.2)	58	(21.4)	12.6	(7.6–20.8)	105	(38.7)	18.2	(11.7–28.1)
Gender*																
Male	22	(1.7)	1		10	(0.8)	1		77	(5.8)	1		109	(8.2)	1	
Female	54	(2.7)	1.2	(0.6–2.1)	22	(1.1)	1.6	(0.7–3.8)	155	(7.7)	1.7	(1.2–2.3)	231	(11.4)	1.6	(1.2–2.2)
**Illiterate**#																
Yes	47	(3.6)	1		12	(1.2)	1		101	(7.8)	1		163	(12.5)	1	
No	29	(1.4)	0.6	(0.4–1.1)	17	(0.8)	1.4	(0.6–3.1)	131	(6.4)	1.2	(0.9–1.6)	177	(8.6)	1.0	(0.8–1.4)
**Marital status**^																
Couple	40	(1.4)	1		18	(0.6)	1		174	(6.0)	1		232	(8.1)	1	
Single	36	(7.7)	2.4	(1.4–4.0)	14	(3.0)	2.0	(1.0–4.4)	58	(12.4)	1.1	(0.7–1.5)	108	(23.0)	1.5	(1.1–2.0)

VI: Visual Impairment, was defined as presenting visual acuity <6/18 and≥6/60; SVI: Severe Visual Impairment, defined as presenting visual acuity <6/60 and ≥3/60; OR: Odds Ratio; CI: Confidence Interval;

^+^: Adjusted by gender, education and marital status;

*: Adjusted by age, education and marital status;

^**#**^**:** Adjusted by age, gender and marital status;

**^:** Adjusted by age, gender and education

### Causes of blindness and visual impairment

Totally, 11 subjects were advised to attend the clinic for further examination when definitive diagnosis could not be made at their houses. Table 4 shows the percentages of major causes of visual loss. Cataract was the leading cause of blindness (67.1%), followed by posterior segment disorders (11.8%), and then refractive error (7.1%). Overall, cataract was the main cause of any visual impairment (PVA<6/18) (48.8%), followed by refractive error (33.2%), and then posterior segment diseases (11.5%). Of all causes of blindness, 83.5% were considered to be avoidable, of which 75.3% were curable and 8.2% treatable. Similarly, 75.7% of SVI and 88.5% of VI were avoidable.

**Table 4:** Degree and cause of presenting visual loss by person.

	Blindness		SVI		VI		Any visual impairment	
Cause	n	(%)	n	%	n	%	n	%
**Refractive error**	6	(7.1)	1	(2.7)	114	(46.9)	121	(33.2)
**Cataract**	57	(67.1)	25	(67.6)	96	(39.5)	178	(48.8)
**Aphakia**	1	(1.2)	0	(0.0)	1	(0.4)	2	(0.5)
**Surgical complications**	2	(2.4)	1	(2.7)	1	(0.4)	4	(1.1)
**Corneal scar**	3	(3.5)	1	(2.7)	2	(0.8)	6	(1.6)
**Glaucoma**	3	(3.5)	0	(0.0)	0	(0.0)	3	(0.8)
**Diabetic retinopathy**	1	(1.2)	0	(0.0)	3	(1.2)	4	(1.1)
**Age-Related Macular Degeneration**	0	(0.0)	0	(0.0)	2	(0.8)	2	(0.5)
**pterygium**	2	(2.4)	0	(0.0)	1	(0.4)	3	(0.8)
**Other posterior segment or CNS disorder**	10	(11.8)	9	(24.3)	23	(9.5)	42	(11.5)
**Total**	85	(100.0)	37	(100.0)	243	(100.0)	365	(100.0)

SVI: Severe Visual Impairment; VI: Visual Impairment

### Outcomes of cataract surgery

In this survey, 155 out of 3484 (4.4%) people (211 eyes) had cataract surgery. Among all the operated patients, 56 (36.1%) received bilateral surgery. Of the 211 eyes that received cataract surgery, 96.7% had an intraocular lens (IOL) inserted. Thirty-five (16.6%) eyes had poor outcome with a PVA of worse than 6/60. The proportion decreased to 13.8% with pin-hole vision. Causes for post-operative VA <6/18 included surgery complications in 19 eyes (27.5%), co-morbidities in 29 eyes (42.0%), long-term complications (subsequent vision loss due to post-operative capsule opacification or retinal detachment) in 4 eyes (5.8%), and un-corrected refractive error in 17 eyes (24.6%). Table 5 compares the outcomes of surgery from different surgical locations. Cataract operations performed in the charity centers resulted in a higher rate of IOL insertion, better visual outcomes (VA≥6/18) and a lower surgical complication rate compared with the local clinics.

**Table 5:** Outcomes of cataract surgery in 211 eyes.

	Total (n = 211)		Place of cataract operation					
			PH (n = 41)		CEC (n = 131)		PC (n = 39)	
	*No*.	(%)	*No*.	(%)	*No*.	(%)	*No*	(%)
**Lens status**								
Aphakia	7	(3.3)	2	(4.9)	1	(0.8)	4	(10.3)
Pseudophakia	204	(96.7)	39	(95.1)	130	(99.2)	35	(89.7)
**Presenting Visual Acuity**								
Normal (PVA≥6/18)	142	(67.2)	30	(73.2)	94	(71.7)	18	(46.1)
VI (PVA <6/18 and ≥6/60)	34	(16.1)	4	(9.8)	21	(16.0)	9	(23.1)
SVI (PVA<6/60 and ≥3/60)	9	(4.3)	1	(2.4)	6	(4.58)	2	(5.1)
Bindness (PVA<3/60)	26	(12.3)	6	(14.6)	10	(7.63)	10	(25.6)
**Pin-hole Visual Acuity**								
Normal (VA≥6/18)	158	(74.9)	31	(75.6)	107	(81.7)	20	(51.3)
VI (VA <6/18 and ≥6/60)	24	(11.4)	3	(7.3)	12	(9.1)	9	(23.1)
SVI (VA<6/60 and ≥3/60)	8	(3.8)	1	(2.4)	4	(3.1)	3	(7.7)
Bindness (VA<3/60)	21	(10.0)	6	(14.6)	8	(6.1)	7	(17.9)
**Causes of PVA <6/18**								
Ocular co-morbidities	29	(13.7)	4	(9.8)	16	(12.2)	9	(23.8)
Surgical complications	19	(9.0)	4	(9.8)	8	(6.1)	7	(17.9)
Uncorrected refractive error	17	(8.1)	2	(4.9)	12	(9.2)	3	(7.7)
Long term complications	4	(1.9)	1	(2.4)	1	(0.8)	2	(5.1)

PVA: Presenting visual acuity; VA: Visual impairment; VI: Visual impairment; SVI: Severe visual impairment; PH: Public Hospitals; CEC: Charity Eye Centers; PC: Private Clinics

### Cataract surgical coverage

Based on the number of operated eyes, the cataract surgical coverage was 51.1% for pinhole VA <3/60; 47.5% for pinhole VA<6/60; and 30.9% for pinhole VA<6/18. In terms of cataract surgical coverage by person, the rates increased to 74.9%, 68.9%, and 47.5% for pin-hole VA <3/60, <6/60 and <6/18 respectively.

### Cataract barriers

The 364 participants with un-operated cataract were interviewed to identify for not having cataract surgery. The fact that they did not know their visual impairment was due to cataract was the main reason (271, 74.5%). Twenty-seven (7.4%) subjects claimed that they did not seek surgery because of old age. Twenty (5.5%) subjects mentioned that the cost of surgery was not affordable. A minority of the participants (46, 1.3%) refused to undergo cataract surgery for other reasons such as fear of surgery, associated medical problems, and so on (S2 Table).

## Discussion

The prevalence of blindness varies in different regions of China, ranging from 0.6% (in Gaungzhou City) to 4.4% (in Hainan province)[2–5, 16, 22–24]. The reported prevalence of blindness in Chaonan in 2012(2.4%) was similar to the Nine-Province survey in China (2.3%) in 2008[16]. The prevalence of blindness in Chaonan (2.4%) was much higher than that in Guangzhou City (0.6%)[3], the capital of Guangdong Province. This may be primarily related to lower socioeconomic status, which has been associated with higher prevalence of blindness[25], among the sample population. Chaonan’s per capita gross domestic product in 2010 was 14,201 RMB (renminbi, Chinese currency unit; approximately USD 2219; 6.4 renminbi = US$1), which was considerably lower than that of Guangdong Province as a whole (43,596 RMB, USD6812) and Guangzhou City (83,494 RMB, USD 13045) in the same year[26]. Similar results were reported in Beijing Eye Study.[27]

In the current study, the prevalence of blindness was higher in females and those who were illiterate. These findings were also observed in other regions of China[16] and other countries including Pakistan[28] and Sudan[29]. The participants who were widowed or unmarried were particularly affected by blindness. This might implicate that family support is very important for patients to seek eye care services. Age was strongly associated with any visual impairment (PVA<6/18) and the risk increased almost 18 times from age of 50 years to age of 80 years. With the increase in the life expectancy in China due to improved socioeconomic conditions and better access to health care services, the prevalence of any visual impairment (PVA<6/18) is likely to increase in the future if no affordable solutions are available.

The large majority (83.5%) of blindness that was detected in this study, was avoidable and cataract was the leading cause, which was consistent with other studies in China[4, 5, 22] and other developing countries[19, 28, 30–33]. Although more than 60% of China’s population lives in these rural regions, more than 80% of the country’s health resources are concentrated in its large cities[10]. Strengthening rural cataract services would be the most effective way to reduce cataract blindness in China. However, most of experienced cataract surgeons in China are concentrated in the urban public hospitals. The number of qualified cataract surgeons in rural areas is less than 2 per million population[10]. The lack of sufficient training opportunities for junior ophthalmologists has adversely limited the manpower for cataract surgery in China. Some rural “eye doctors” without any special training have opened private clinics providing low qualified cataract surgery. The previous population based studies in Doumen and Shunyi[34, 35] have documented poor outcomes of cataract surgeries performed in these rural areas of China, with only 25.0% and 23.7% of the operated eyes having PVA better than 6/18, respectively.

In our study, 46.1% of patients operated in local rural eye clinics had postoperative PVA that was 6/18 or better, which is similar to that reported in the recent Nine-Province survey in China (46.5%)[36]. But better visual outcomes and less surgical complication rates were observed among subjects whose cataract surgery was performed in charitable eye centers. The good surgical outcome (PVA≥6/18, 71.7%) is similar to the metropolitan cities with top medical resources in mainland China. In Beijing[37] and Guangzhou[38], 80% and 62% of cataract-operated eyes had presenting VA > = 6/18. This may be attributed to the well-established quality control system and surgical training procedures implemented in the charity centers. Consultant ophthalmologists from urban centers provided on-site surgical training to local doctors, and supervised on sutureless large incision manual cataract extraction (SLIMCE)[11] [12]. After 6 months of hands-on training, these local surgeons were able to perform cataract surgery independently with encouraging outcomes. Recently, experience from Guangdong province[39] and in Jiangxi[40] have demonstrated that rural trainees were capable of operating independently and achieved good results after appropriate hands-on training[12, 41]. As the favorable outcomes became recognized by residents in the catchment area, the number of patients receiving cataract surgery in charity centers steadily increased. In our study, The prevalence of cataract surgery in Chaonan (4.4%, 95%CI, 3.8%-5.1%) was higher than that reported in Yangjiang County, a rural county of the Guangdong province(3.07%, 95% CI, 2.45%-3.70%), overall reported in the China Nine-Province survey (2.09%)[36] and similar to that in the developed provincial cities including Guangzhou City(4.4%)[38] and Singapore (4.2%)[42].

Currently, the indication for cataract surgery was largely accepted as VA less than 6/18[22, 43]. The cataract surgery coverage was 30.9% for pin-hole VA<6/18. In other words, about 70% of operable cataract did not receive cataract surgery even in this region wherein two charity eye centers were established and two large public hospitals were within 5-hour driving distance. Among the barriers to cataract surgery, lack of awareness their VI due to cataract is the most common reason (74.5%); followed by no perceived need and lack of financial support for surgery. Grounded on this information, the barriers of cataract surgery in this region might be improved by an enhanced screening program and public education. Further studies are needed to identify the most effective measure to promote cataract surgery uptake.

Efficient and effective methods to estimate blindness are vital to policy makers for resource allocation. One of the strengths of the survey is the high response rate, which is important in minimizing selection bias. A population-based survey with 94.1% participation highlights the importance of door-to-door visits, advocacy before the survey and repeated household visits to seek participation. While the coverage of the sample population was high (94.2%), there was a slightly lower response rate in younger males (aged 50–59 years), which was similar to most other population-based surveys[2, 5, 20, 22], The younger people who were absent from home or reluctant to participate might be migrant workers or those with better visual function.

In conclusion, the prevalence of blindness in Chaonan was still high in this region. Cataract blindness is still and will continue to be a heavy burden for the aging population in China. Our study showed that there is significant room for improvement in the quality of surgeries performed in the rural private clinics. The rural cataract surgical services were suboptimal and should be strengthened. Outcomes of cataract surgery in this survey have demonstrated that rural trainees are capable of operating independently and achieving good results after appropriate hands-on training. Appropriate strategies of public awareness programs and patient education might help to improve uptake of cataract surgery and increase cataract surgical coverage.

## Supporting information

S1 Table The age and gender distribution of 134 participants for refusing to answer literacy and marital status. (DOCX)

S2 Table Barriers to uptake of cataract surgery (among subjects with pin-hole VA <6/18 in one or both eyes, with principal cause of as cataract). *A participant could make at most two Reponses.
(DOCX)
