# Supplementary material for: Prevalence of visual impairment and outcomes of cataract surgery in Chaonan, South China
Source: PLoS One. 2017 Aug 10;12(8):e0180769. doi: 10.1371/journal.pone.0180769 (PMC5552304; doi:10.1371/journal.pone.0180769)
Supplement: S1 Table — (DOCX) [file pone.0180769.s001.docx]

**S1 Table. The age and gender distribution of 134 participants for refusing to answer literacy and marital status**

| **Age+ (yrs)** | **Gender** | | | | **Total** | |
| --- | --- | --- | --- | --- | --- | --- |
|  | Male | | Female | |  |  |
|  | *No.* | *(%)* | *No.* | *(%)* | *No.* | *(%)* |
| 50-59 | 31 | (44.9) | 19 | (29.2) | 50 | (37.3) |
| 60-69 | 18 | (26.1) | 22 | (33.8) | 40 | (29.9) |
| 70-79 | 11 | (15.9) | 10 | (15.4) | 21 | (15.7) |
| >80 | 9 | (13.0) | 14 | (21.5) | 23 | (17.2) |
| **Total** | 69 | (110.0) | 65 | (100.0) | 134 | (100.0) |
