# Supplementary material for: Prevalence of visual impairment and outcomes of cataract surgery in Chaonan, South China
Source: PLoS One. 2017 Aug 10;12(8):e0180769. doi: 10.1371/journal.pone.0180769 (PMC5552304; doi:10.1371/journal.pone.0180769)
Supplement: S2 Table — *A participant could make at most two Reponses. (DOCX) [file pone.0180769.s002.docx]

**S2 Table. Barriers to uptake of cataract surgery** (among subjects with pin-hole VA <6/18 in one or both eyes, with principal cause of as cataract)

| **Reasons given for not having cataract surgery** | **n** | **%** |
| --- | --- | --- |
| Do not know they have cataract | 271 | 74.5 |
| Do not know cataract is treatable | 1 | 0.3 |
| Do not believe treatment is effective | 0 | 0.0 |
| Believes it to be destiny / God's Will | 1 | 0.3 |
| Told to wait for cataract to mature | 7 | 1.9 |
| Surgical services not available or very far | 1 | 0.3 |
| Don't know how to get surgery | 3 | 0.8 |
| Cannot afford operation | 20 | 5.5 |
| No one to accompany | 4 | 1.1 |
| No time available / other priorities | 1 | 0.3 |
| Old age and need not felt | 27 | 7.4 |
| One eye adequate vision / need not felt | 17 | 4.7 |
| Fear of operation | 5 | 1.4 |
| Fear of losing eye sight | 1 | 0.3 |
| Other disease contra-indicating operation | 5 | 1.4 |
| **Total*** | **364** | **100.0** |

*A participant could make at most two Reponses
